# Transient Receptor Potential Channel A1 (TRPA1) Regulates Sulfur Mustard-Induced Expression of Heat Shock 70 kDa Protein 6 (*HSPA6*) In Vitro

**DOI:** 10.3390/cells7090126

**Published:** 2018-08-31

**Authors:** Robin Lüling, Harald John, Thomas Gudermann, Horst Thiermann, Harald Mückter, Tanja Popp, Dirk Steinritz

**Affiliations:** 1 Bundeswehr Institute of Pharmacology and Toxicology, Ludwig-Maximilians-Universität Munich, 80937 Munich, Germany; robin1lueling@bundeswehr.org (R.L.); haraldjohn@bundeswehr.org (H.J.); horstthiermann@bundeswehr.org (H.T.); tanjapopp@bundeswehr.org (T.P.); 2 Walther-Straub-Institute of Pharmacology and Toxicology, Ludwig-Maximilians-Universität Munich, 80336 Munich, Germany; thomas.gudermann@lrz.uni-muenchen.de (T.G.); Mueckter@lrz.uni-muenchen.de (H.M.)

**Keywords:** 2D gel electrophoresis, AP18, HEK293, HSP70, MALDI-TOF MS(/MS), nanoHPLC-ESI MS/MS, proteomics, sulfur mustard, TRPA1

## Abstract

The chemosensory transient receptor potential ankyrin 1 (TRPA1) ion channel perceives different sensory stimuli. It also interacts with reactive exogenous compounds including the chemical warfare agent sulfur mustard (SM). Activation of TRPA1 by SM results in elevation of intracellular calcium levels but the cellular consequences are not understood so far. In the present study we analyzed SM-induced and TRPA1-mediated effects in human TRPA1-overexpressing HEK cells (HEKA1) and human lung epithelial cells (A549) that endogenously exhibit TRPA1. The specific TRPA1 inhibitor AP18 was used to distinguish between SM-induced and TRPA1-mediated or TRPA1-independent effects. Cells were exposed to 600 µM SM and proteome changes were investigated 24 h afterwards by 2D gel electrophoresis. Protein spots with differential staining levels were analyzed by matrix-assisted laser desorption/ionization time-of-flight mass spectrometry and nano liquid chromatography electrospray ionization tandem mass spectrometry. Results were verified by RT-qPCR experiments in both HEKA1 or A549 cells. Heat shock 70 kDa protein 6 (*HSPA6*) was identified as an SM-induced and TRPA1-mediated protein. AP18 pre-treatment diminished the up-regulation. RT-qPCR measurements verified these results and further revealed a time-dependent regulation. Our results demonstrate that SM-mediated activation of TRPA1 influences the protein expression and confirm the important role of TRPA1 ion channels in the molecular toxicology of SM.

## 1. Introduction

The chemical warfare agent sulfur mustard (SM) causes severe damage to the skin, eyes, and the respiratory system [1,2]. Although SM and the associated injuries have been intensively investigated over decades, the molecular toxicology is still not understood in detail. In aqueous environments, SM forms a highly reactive sulfonium and subsequent carbenium ion [3]. A plethora of nucleophiles including the N7 atom of guanine bases in the DNA helix are targeted by SM. Monofunctional DNA alkylation and in particular DNA crosslinks were regarded as the exclusive mechanism of toxicity. However, Stenger et al. demonstrated that the alkylating substances CEES (2-chloroethyl-ethyl sulfide, a mono-functional SM analogue) and SM activate transient receptor potential ankyrin 1 cation channels (TRPA1) in vitro, thereby affecting cell viability [4].

TRPA1 channels belong to the TRP channel superfamily and are located in the plasma membrane of different human cell types, predominantly of neuronal cells [5].

They usually form homotetramers, but heterotetramers with TRPV1 have also been described [6,7]. TRP channels share the overall architecture of voltage-gated ion channels with six transmembrane domains (TMs). TM5 and TM6 form the pore region that is permeable for monovalent K^+^, Na^+^ and bivalent Ca^2+^ or Mg^2+^ cations [8]. The intracellular N-terminus of TRPA1 possesses multiple characteristic ankyrin repeat domains that contain free cysteine residues that are important for channel activity [9]. The physiological function of TRPA1 is the perception of sensory stimuli like pain and cold but also of certain reactive chemicals such as acrolein, a highly reactive substance present in tear gas or vehicle exhausts [10,11,12]. The activation of TRPA1 by reactive compounds is assumed to rely on covalent modification of cysteine residues in the ankyrin repeat sequence [9,10,12]. Reactive oxygen species (ROS), hypochlorite and protons were also identified as TRPA1 activators [13,14,15,16,17,18]. The latter seem to interact with an extracellular interaction site of TRPA1 and not via modification of intracellular cysteines [13].

The highly reactive SM and CEES were also identified as distinct TRPA1 activators with a not yet identified binding site [4,19]. Both chemicals provoked a TRPA1-dependent increase of intracellular calcium levels ([Ca^2+^]_i_) that could be efficiently prevented by pre-incubation with the TRPA1-specific blocker AP18 [4,19]. There is some evidence that TRPA1 activation is involved in the molecular toxicity of alkylating compounds [4,20,21]. However, the cellular consequences of an SM-induced and TRPA1-mediated elevation of [Ca^2+^]_i_ have not been investigated in detail so far. In the present study we analyzed TRPA1-dependent effects after SM exposure in human TRPA1-overexpressing HEK cells (HEKA1). Proteome changes were analyzed by 2D gel electrophoresis (2D-GE) with subsequent matrix-assisted laser desorption/ionization time-of-flight mass spectrometry (MALDI-TOF MS(/MS)) and nano high performance liquid chromatography electrospray ionization tandem mass spectrometry (nanoHPLC-ESI MS/MS). AP18 was used to distinguish between TRPA1-dependent and -independent effects on protein expression. Results were validated by RT-qPCR in HEKA1 cells and as well as in human A549 lung epithelial cells endogenously expressing TRPA1 channels [22,23,24].

## 2. Materials & Methods

### 2.1. Chemicals

SM was made available by the German Ministry of Defense and integrity as well as purity was proved by NMR in house. Allylisothiocyanat (AITC), iodoacetamid (IAA), ethanol (EtOH), glycerol, glycine, trifluoroacetic acid (TFA), acetonitrile (ACN), penicillin/streptomycin (P/S) and Trypsin Profile IGD Kit for proteolysis of protein spots were obtained from Sigma-Aldrich (Steinheim, Germany). 2D-Clean-UP Kit, Silver Staining Kit (protein), 2D-Quant-Kit and Coomassie Brilliant Blue Solution were obtained from GE Healthcare (Freiburg, Germany). Bromophenol blue and sodium dodecyl sulfate (SDS) were purchased from Bio-Rad (Munich, Germany). RT^2^ First Strand Kit, RT^2^ SYBR Green/ROX qPCR Mastermix, RNeasy Protect Mini Kit and RT^2^ Custom Profiler PCR 96-well plates with specific customized primers were purchased from QIAGEN Sciences (Venlo, The Netherlands). Dulbecco’s minimal Eagle medium (DMEM), fetal bovine serum (FBS), trypsin–EDTA (ethylenediaminetetraacetic acid) and phosphate-buffered saline (PBS) were obtained from Life Technologies (Gibco, Karlsruhe, Germany). AP18 was delivered by Bio-Techne (Wiesbaden-Nordenstadt, Germany). α-cyano-4-hydroxycinnamic acid (CHC) as matrix for MALDI-TOF measurements was obtained from Bruker Daltonics (Bremen, Germany).

### 2.2. Cell Culture

HEK293 wild-type cells (introduced as HEKwt) and HEK293-A1-E cells (introduced as HEKA1) with a stable expression of human TRPA1 (hTRPA1) were kindly donated by the Walther-Straub-Institute of Pharmacology and Toxicology (Ludwig-Maximilians-Universität, Munich). Cells were grown in DMEM containing 4.5 g/L glucose, Earl’s salts and L-glutamine. This medium was supplemented with 10% FBS (*v*/*v*) and 1% P/S (*v*/*v*). Cells were cultured in a humidified atmosphere at 5% (*v*/*v*) CO_2_ and 37 °C (standard conditions). HEKwt cells were split every 2–3 days while HEKA1 cells were subcultivated every 3–4 days. Cells were detached using trypsin-EDTA for 3 min and resuspended in the respective medium. A549 cells were grown in DMEM (Biochrom, Berlin, Germany) supplemented with FBS (Biochrom, Berlin, Germany) and gentamycin (5 µg/mL). Cells were split every 2–3 days detached by trypsin-EDTA for 5 min.

### 2.3. Sample Preparation

HEKwt, HEKA1 or A549 cells were exposed to 600 µM SM according to Stenger et al. [4,19]. A concentration of 25 µM AITC was used to stimulate HEKwt and HEKA1 cells. Cell lysates of SM-exposed or AITC-treated cells were generated at 24 h for 2D-GE. Pre-incubation with the TRPA1-specific inhibitor AP18 (2 µM, application 5 min prior to SM or AITC exposure) was also performed according to Stenger et al. for all groups [4]. Controls were incubated without AITC or SM but with medium and, if applicable, with AP18. After the respective incubation time, cells were washed with PBS first and then harvested with trypsin-EDTA for 3 min and resuspended in 10 mL DMEM. Cell number was determined using a Neubauer counting chamber (NanoEnTek, Seoul, Korea). Cells were lysed in lysis buffer (7 M urea, 2 M thiourea, 4% *w*/*v* CHAPS, 2% *v*/*v* IPG buffer, 40 mM DTT) for 2D-GE. Samples were sonicated (4 cycles with 10 s) on ice. Supernatants were collected after centrifugation (30 min, 4 °C and 21,130 RCF) and subsequently cleaned up using the 2D Clean-Up Kit according to the manual of the provider. Protein concentration was determined using the 2D-Quant Kit. Samples were aliquoted and frozen at −80 °C.

For RT-qPCR, HEKA1 cells were investigated 1, 3, 5, or 24 h after exposure while HEKwt and A549 cells were analyzed after 24 h only. Approx. 10 × 10^6^ cells were collected in 1 mL of RNA protection reagent from QIAGEN (Hilden, Germany) at the respective time points. RNA was extracted using the RNeasy Mini Protect Kit (QIAGEN, Hilden, Germany) according to the instructions given by the manufacturer. In brief, cell pellets were lysed in 600 µL RLT lysis buffer and homogenized using a QIA shredder (QIAGEN). RNA was precipitated in 600 µL 70% (*v*/*v*) EtOH and purified by washing several times in different buffers according to the manufacturer’s protocol. The concentration of RNA was measured using the NanoDrop 8000 Spectrophotometer from Thermo Scientific (Schwerte, Germany).

### 2.4. 2D Gel Electrophoresis and Image Analysis

IPG strips (Immobiline Drystrips 7 cm, pH 4–7 or pH 6–11, linear, or pH 3-11 non-linear, GE Healthcare, Chicago, IL, USA) were rehydrated with 8 M urea, 2% (*w*/*v*) CHAPS, 0.5% (*w*/*v*) DTT, 0.5% (*v*/*v*) IPG buffer together with 60 µg for pH 4–7 strips, 150 µg for pH 6–11 strips or 6 µg for pH 3–11 strips of the protein lysates. Following rehydration loading for approx. 17 h in an Immobiline DryStrip IPGbox (GE Healthcare), first dimension isoelectric focusing (IEF) was performed using an Ettan IPGphor II (GE Healthcare) for 8 kVh at a maximum voltage of 5000 V and a limiting current of 50 µA/strip. Afterwards, gel strips were equilibrated in 10 mg/mL DTT equilibration buffer (6 M urea, 75 mM Tris-HCl pH 8.8, 29.3% *v*/*v* glycerol, 2% *w*/*v* SDS) for 15 min and afterwards in 25 mg/mL IAA equilibration buffer for further 15 min. Strips were transferred on 10% Bis-Tris gels (Thermofisher Scientific, Waltham, MA, USA), sealed with agarose sealing solution (25 mM Tris base, 192 mM glycine, 0.1% *w*/*v* SDS, 0.5% *w*/*v* agarose, 0.002% *w*/*v* bromophenol blue) and separated in a second dimension according to electrophoretic mobility with constant voltage of 180 V for 1 h (SDS-PAGE). Proteins separated by pH 4–7 strips or pH 6–11 strips were then stained with colloidal Coomassie Brilliant Blue (CBB, GE Healthcare) for 35 min and scanned on a Microtek Bio-5000 scanner (Serva, Heidelberg, Germany). Proteins separated on pH 3–11 strips were stained using the Silver Staining Kit (GE Healthcare). In short, gels were soaked in fixing solution (30% *v*/*v* EtOH, 10% *v*/*v* glacial acetic acid) for 60 min and then sensitized in sensitizing solution (30% *v*/*v* EtOH, 5% *w*/*v* sodium thiosulphate, 6.8% *w*/*v* sodium acetate) for a further 60 min. After washing with distilled water for four times, silver solution (2.5% *w*/*v* silver nitrate solution) was added for 60 min. A developing solution (2.5% *w*/*v* sodium carbonate, 37% *w*/*v* formaldehyde) was added until spots reached desired intensity. Then, gels were transferred to a stopping solution (1.5% *w*/*v* EDTA-Na_2_) before a preserving solution (30% EtOH, 87% *w*/*w* glycerol) was added. Gels were scanned on a Microtek Bio-5000 scanner (Serva, Heidelberg, Germany).

Protein spots with significant different staining levels were identified using Progenesis SameSpots software v5.0.0.7 (Nonlinear Dynamics, Newcastle, UK). Threshold levels were defined with a fold change > 2.0 and an ANOVA *p* value < 0.05. Spots were filtered to identify only those which applied to both criteria. At least 3 biological replicates were investigated for each group. EtOH solvent control gels were chosen as reference.

### 2.5. MALDI-TOF MS(/MS) or NanoHPLC-ESI MS/MS Analysis

Relevant protein spots were excised and proteolyzed in-gel using the trypsin profile IGD kit (Sigma-Aldrich). In brief, the gel piece was covered with 200 µL destaining solution and incubated at 37 °C for 30 min. The gel piece was dried before 20 µL (0.4 µg of trypsin) of the prepared trypsin solution and 50 µL of the trypsin reaction buffer were added. It was incubated overnight at 37 °C. Following tryptic cleavage, peptides were desalted and concentrated using ZipTip-C18 pipette tips (Merck Millipore, Darmstadt, Germany). First, ZipTip was equilibrated using 10 µL methanol and 10 µL 0.1% (*v*/*v*) TFA. Afterwards, sample was loaded by pipetting the digested protein up and down for 10 times. ZipTip was washed with 10 µL 0.1% (*v*/*v*) TFA before sample was eluted with 10 µL of acetonitrile/0.1% (*v*/*v*) TFA (80/20 *v*/*v*). Using the dried-droplet technique, samples were spotted onto a polished steel target by mixing 1 µL each of sample and CHC (5 mg/mL in a 1:2 mixture of ACN and 0.1% *v*/*v* TFA).

MALDI-TOF MS(/MS) measurements were performed in the positive reflector ion mode using an Autoflex III smartbeam mass spectrometer (Bruker, Billerica, MA, USA) equipped with a modified pulsed all-solid-state laser 355 nm (Bruker Daltonics). A peptide mass fingerprint (PMF) was recorded in a mass range from *m*/*z* 900–3400 with the following settings: Ion source I, 19 kV; ion source II, 16.5 kV; lens, 8.3 kV; reflector I, 21 kV; reflector II, 9.75 kV. MS/MS experiments were executed in the LIFT mode with the following parameters: Ion source I, 6 kV; ion source II, 5.3 kV; lens, 3.0 kV; reflector I, 27 kV; reflector II, 11.6 kV; LIFT I, 19 kV; LIFT II, 4.2 kV.

The mass spectrometer was calibrated using the peptide standard mixture of bradykinin (1–7), angiotensin II, angiotensin I, substance P, bombesin, renin substrate, ACTH clip (1–17), ACTH clip (18–39) and somatostatin (peptide calibration standard II, Bruker Daltonics).

Mass spectra were recorded using the flex control software v.3.0 (Bruker, Billerica, MA, USA) and further processed by flex analysis v.3.0 and BioTools v.3.1.2.22 (both Bruker). Identification of proteins was achieved via the SwissProt protein database using MS ion search of the Mascot search engine (Matrix Science, London, England) with following search criteria: Taxonomy Homo sapiens (human), enzyme trypsin, fragment mass tolerance 0.1%, significance threshold *p* < 0.05, maximum number of hits 20.

Protein spots that could not be identified by MALDI-TOF MS(/MS) were analyzed by more sensitive nanoHPLC-ESI MS/MS (proteome factory AG, Berlin, Germany). The LC MS/MS system consisted of an Agilent 1100 nanoHPLC system (Agilent, Waldbronn, Germany), PicoTip electrospray emitter (New Objective, Woburn, MA, USA) and an Orbitrap XL mass spectrometer (ThermoFisher Scientific, Bremen, Germany). Peptides were first trapped and desalted on the enrichment column (Zorbax 300SB-C18, 0.3 × 0.5 mm, Agilent, Santa Clara, CA, USA) for five minutes (solvent: 2.5% ACN/0.5% formic acid). Then, they were separated on a Zorbax 300SB-C18, 75 µm × 150 mm column (Agilent) using a linear gradient from 15% to 40% B (solvent A: 0.1% formic acid in water, solvent B: 0.1% formic acid in ACN). Ions of interest were data-dependently subjected to MS/MS according to the expected charge state distribution of peptide ions. MS/MS data were matched against the SwissProt protein database using MS/MS ion search of the Mascot search engine (Matrix Science, London, UK) with following parameters: Enzyme trypsin, fixed modifications carbamidomethyl (C), variable modifications deamidated (NQ) and oxidation (M), mass values monoisotopic, peptide mass tolerance 3 ppm, fragment mass tolerance 0.6 Da, significance threshold *p* < 0.05, taxonomy Homo sapiens (human).

### 2.6. Real-Time qPCR

Extracted RNA (500 ng) was transcribed into complementary DNA (cDNA) using the RT^2^ First Strand Kit. Transcription was performed according to the manufacturer’s protocol. In brief, 10 µL of a reverse transcriptase mixture was added to the RNA samples. The mixture was incubated for 15 min at 42 °C and then for another 5 min at 95 °C. From each resulting cDNA sample, 675 µL were mixed with 675 µL of RT^2^ SYBR Green/ROX qPCR Mastermix. A volume of 25 µL from each sample was transferred into a specially designed RT^2^ Custom Profiler PCR 96-well plate using a TECAN freedom evo (TECAN, Crailsheim, Germany). 96-well plates were pre-spotted with specific primers according to the results of 2D-GE. Plates were sealed with cap s–trips and placed into the Mastercycler 2S (Eppendorf, Hamburg, Germany). The qPCR was carried out with the following PCR program: 10 min at 95 °C followed by 40 cycles of 15 s at 95 °C, and 1 min at 60 °C. At the end of the PCR program, a melting profile of the DNA amplifications was measured with the following settings: 95 °C for 15 s, 60 °C for 15 s and a final temperature gradient from 60 °C to 95 °C over 20 min. PCR data were analyzed with the realplex software from Eppendorf and with an online software from QIAGEN [25].

## 3. Results

### 3.1. 2D Gel Electrophoresis and Mass Spectrometry

**Figure 1:**
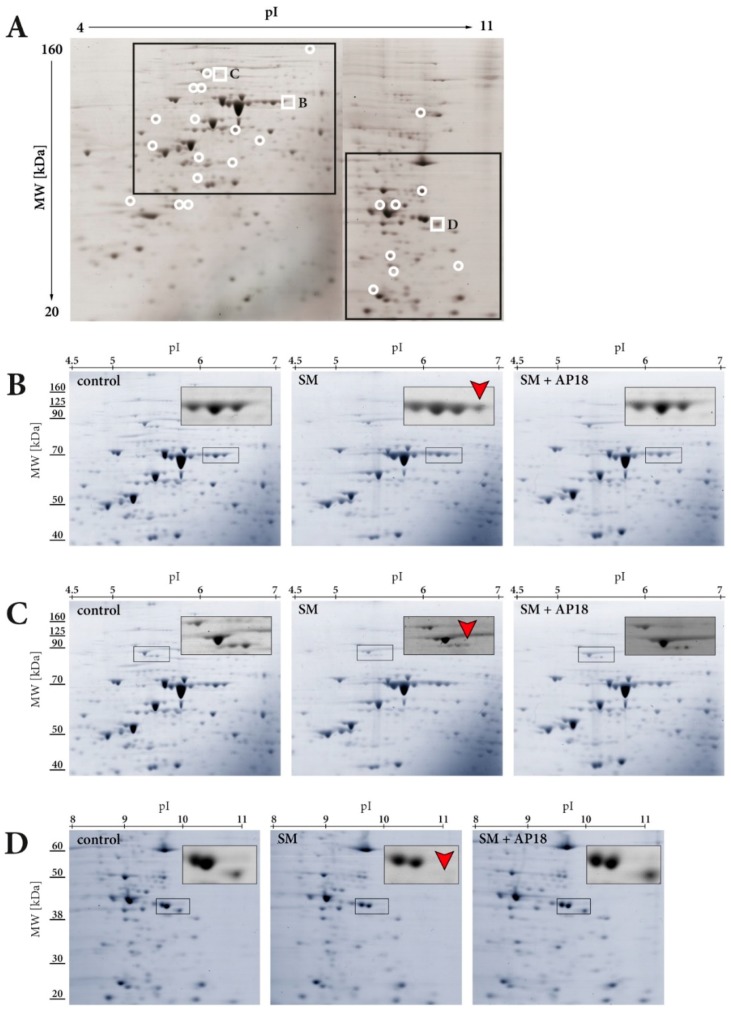
Representative 2D CBB-stained gel electrophoresis of HEKA1 cells. The proteome of HEKA1 control, 600 µM SM-exposed or 2 µM AP18-pre-incubated and SM-exposed cells was investigated. Isoelectric focusing of cell lysates was performed by 7 cm strips (pH 4–7 and pH 6–11, linear). Proteins were separated by 10% Bis-Tris gels. (**A**) Overview gel displaying 22 differential protein spots after SM exposure compared to controls (white open circles). White frames indicate three SM-induced and TRPA1-regulated proteins. (**B**) Zoom of the *HSPA6* spot and (**C**,**D**) zoom of the two protein spots which proteins are listed in Table 1. Experiments were carried out with *n* = 3 per group. Molecular weight is indicated on the left and the pI value on top of the gels.

**Figure 2:**
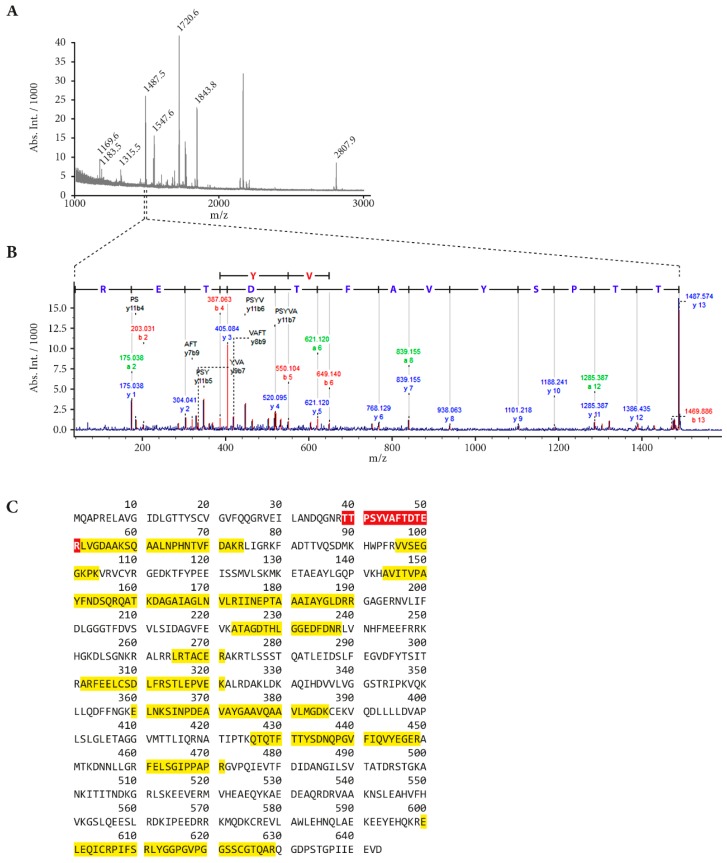
MALDI-TOF MS(/MS) measurements of a protein spot after tryptic cleavage identified HSP6A. (**A**) Peptide mass fingerprint of *HSPA6* identified by MASCOT database matching resulted in a score of 86.6. (**B**) MS/MS spectrum of the ion at *m*/*z* 1487.5 resulted in a sequence tag of 13 amino acids. The complete series of y-ions could be found. (**C**) A sequence coverage of 35.5% was found for *HSPA6* (UniProtKB-P17066). Assigned peptides are indicated with a yellow background. The sequence of the peptide subjected to MS/MS fragmentation depicted in (**B**) (amino acids 39–51) is highlighted with a red background.

Analysis of HEKA1 cells exposed to SM and investigated after 24 h revealed differential detection of 22 protein spots compared to the control group (Figure 1A) in CBB-stained 2D gels. Three of these spots were identified with a threshold level of a fold change > 2.0 together with a *p* value < 0.05 and to be dependent on TRPA1 (Figure 1B–D). Dependency on TRPA1 was proven as pre-incubation with AP18 prevented SM-induced effects. Up-regulation of one (Figure 1B) and down-regulation of two protein spots (Figure 1C,D) were observed. The up-regulated protein was identified by MALDI-TOF MS peptide mass fingerprint (Figure 2A) and subsequent MS/MS analysis of characteristic protein-derived peptides as heat shock 70 kDa protein 6 (*HSPA6*, UniProtKB-P17066). Fragmenting of the ion at *m*/*z* 1487.5 is exemplarily shown in Figure 2B and documents the internal peptide (39–51). The overall sequence coverage was 35.5% (Figure 2C). The Mascot probability score was calculated to be 86.6. Identification of the two down-regulated protein spots was not successful by the MALDI-TOF technique. Therefore, spots were analyzed by the more sensitive nanoHPLC-ESI MS/MS which identified 4 proteins for each spot all with a high probability score. Table 1 gives an overview on the detected proteins. Values for the respective molecular weight were taken from UniProt database.

Silver-stained 2D gels identified 28 additional protein spots (12 up- and 16 down-regulated) that were affected after SM exposure (Figure S1). AP18 pre-incubation did not influence the SM-induced changes, thereby excluding the involvement of TRPA1 (data not shown).

### 3.2. RT-qPCR

**Figure 3:**
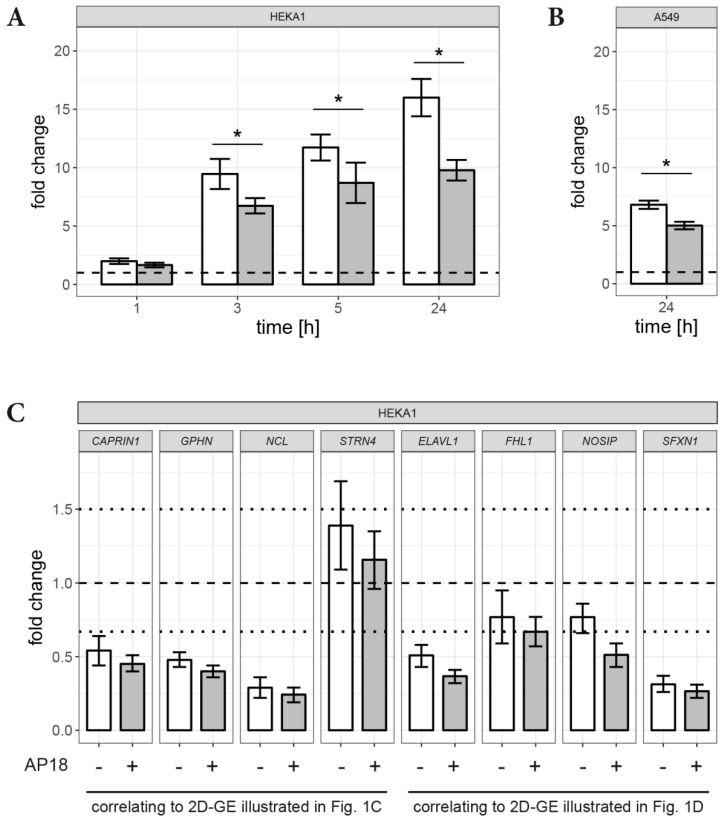
RT-qPCR measurements for potentially SM-affected and TRPA1-regulated genes. mRNA levels of (**A**) *HSPA6* in HEKA1 cells, (**B**) *HSPA6* in A549 cells and (**C**) *CAPRIN1*, *ELAVL1*, *FHL1*, *GPHN*, *NOSIP*, *NCL*, *SFXN1* and *STRN4* in HEKA1 cells were analyzed 24 h after 600 µM SM exposure by RT-qPCR. White bars indicate the fold change values after SM exposure while grey bars illustrate the effect of 2 µM AP18 pre-incubation on mRNA levels. The dashed lines represent normalized levels of the control samples and dotted lines indicate ±1.5-fold change ranges. Significant differences (*p* < 0.05) are displayed by asterisks (*). Error bars represent the 95% confidence intervals. Data are derived from independent biological experiments (*n* = 3). Gene names correspond to the following proteins: Caprin-1 (*CAPRIN1*), ELAV like protein 1 (*ELAVL1*), Four and a half LIM domain protein 1 (*FHL1*), Gephyrin (*GPHN*), Nitric oxide synthase interacting protein (*NOSIP*), Nucleolin (*NCL*), Sideroflexin 1 (*SFXN1*) and Striatin 4 (*STRN4*).

The results of 2D-GE analysis were confirmed by independent RT-qPCR experiments (Figure 3). Accordingly, genes for *HSPA6*, *CAPRIN1*, *ELAVL1*, *FHL1*, *GPHN*, *NOSIP*, *NCL*, *SFXN1* and *STRN4* were chosen as targets. Effects on transcription of these genes were assessed 24 h after SM exposure. In HEKA1 cells, SM significantly increased *HSPA6* mRNA (16.0 × [14.4 − 17.6, 95% CI]) compared to controls (normalized to 1.0) (Figure 3A). AP18 attenuated this up-regulation significantly (9.78 × [8.9 − 10.66, 95% CI]) (Figure 3A). As these results confirmed findings from the 2D-GE, additional time points (1, 3, 5 h and 24 h) were investigated. A minor increase of *HSPA6* mRNA levels was detectable already 1 h after SM exposure (1.99 × [1.75 − 2.23, 95% CI]) (Figure 3A). After 3 h, a more pronounced increase (9.5 × [8.2 − 10.8, 95% CI]) was observed, which further increased after 5 h (11.7 × [10.6 − 12.8, 95% CI]) (Figure 3A). AP18 pre-incubation significantly decreased *HSPA6* mRNA levels after 3 h and beyond (1 h: 1.6 × [1.5 − 1.9]; 3 h: 6.7 × [6.1 − 7.4]; 5 h: 8.7 × [7.0 − 10.4]) (Figure 3A).

AITC treatment, also with AP18 pre-incubation, of HEKA1 was conducted to elucidate the role of TRPA1 activation in more detail (Figure S2). As expected, AITC resulted in a pronounced increase of *HSPA6* mRNA levels 24 h after treatment that was minimized to less than 50% by AP18. AP18 alone without AITC did not affect *HSPA6* mRNA. Also, no changes of *HSPA6* mRNA levels were observed in HEKwt cells after AP18 or SM exposure (Figure S2).

Human A549 cells, endogenously expressing TPRA1, responded with a distinct increase of *HSPA6* mRNA levels after SM exposure measured 24 h after exposure (6.8 × [6.5 − 7.2]) that was significantly diminished by AP18 (5.0 × [4.7 − 5.3]) (Figure 3B).

Levels of *FHL1*, *NOSIP* or *STRN4* mRNA showed some slight SM-induced changes, but levels were not in the range of ±1.5-fold compared to controls. *CAPRIN1*, *ELAVL1*, *GPHN*, *NCL* and *SFXN1* mRNA levels were down-regulated 24 h after SM exposure. However, AP18 was unable to increase these mRNA levels. A summary of the fold change values is given in Table S1.

## 4. Discussion

Cell damage caused by alkylating compounds is assumed to rely on DNA mono-adducts and particularly on DNA crosslinks or the biological consequences thereof [28,29,30]. However, cytotoxic effects of alkylating agents are strongly attenuated by cellular DNA repair processes [29,31,32,33]. Therefore, additional complex mechanisms have been proposed including PARP signaling, nitric oxide and oxidative stress and activation of multiple cellular pathways that contribute to cytotoxicity [28,34,35,36,37,38,39]. In this context, chemosensing TRPA1 channels were described as targets of SM and related alkylating compounds [4,19]. A distinct increase of [Ca^2+^]_i_ occurred after the activation of TRPA1 by SM. Some biological effects thereof, e.g., influence on cell viability, have already been described [4]. Additional SM-induced and TRPA1-mediated effects have not been studied so far and were investigated in this study.

HEKA1 cells, overexpressing human TRPA1 channels, as well as human A549 lung epithelial cells, endogenously expressing TRPA1, were chosen as the in vitro model. Both cell types were used in several studies before and were found very well suited for the investigation of TRPA1-related effects [23,40,41]. Several genes and proteins have been reported to be specifically up-regulated in mouse skin and in human keratinocytes after exposure to SM [42,43]. Thus, we focused on proteome changes after SM exposure with special focus on the involvement of TRPA1.

2D-GE with subsequent protein identification by MS were used to detect changes of protein levels in HEKA1 cells. Cell lysates from controls, which were only treated with the solvent EtOH, were selected as control group. SM-treated or cells pre-incubated with the specific TRPA1 inhibitor AP18 [44], were examined to unambiguously identify SM-induced and TRPA1-regulated proteins.

Our results indicated 22 differentially expressed protein spots after SM exposure in HEKA1 cells compared to un-exposed controls (Figure 1A). It should be noted that we have chosen 7 cm first dimension gel strips covering pH-ranges between 4–7 or 6–11 and proteins were visualized after SDS-PAGE separation by CBB staining. CBB staining detects high-abundant proteins with a very good chance of success for the identification by MALDI-TOF MS(/MS) while changes in low-abundant proteins may be undiscovered. Additional silver staining experiments were conducted and identified 28 further protein spots. However, AP18 pre-incubation had no effect on these spots, thereby excluding a role of TRPA1. Nevertheless, we successfully identified three SM-induced and TRPA1-regulated protein spots 24 h after exposure with one up-regulated and two down-regulated proteins (Figure 1B–D). The up-regulated protein was unequivocally identified as heat shock 70 kDa protein 6 (*HSPA6*) by MALDI-TOF MS peptide mass fingerprint and further MS/MS fragmentation of prominent peptide ions (Figure 2A,B). Identification of the down-regulated protein spots by MALDI-TOF MS was not successful, most probably due to insufficient protein amounts. Therefore, nanoHPLC-ESI MS/MS was chosen as an alternative method. Using this highly sensitive MS/MS method, multiple proteins with high probability scores were unambiguously verified (Table 1). All proteins that were assigned to the respective spot revealed a similar MW and a pI, in line with the 2D-GE results. It is not uncommon in 2D-GE that protein spots, especially of high-abundant proteins, do not represent a single protein. Instead proteins with similar MW and pI can overlap which is also the case in our experiments. The identity of proteins was confirmed by RT-qPCR. SM exposure resulted in down-regulation of all investigated mRNA except *STRN4* (Figure 3C). Some effects were weak and failed to meet the criteria of a ±1.5-fold change (*STRN4*, *FHL1*, *NOSIP*) while *CAPRIN1*, *GPHN*, *NCL* and *ELAVL1* mRNA were down-regulated to some extend (Figure 3C). However, AP18 did not significantly influence mRNA levels in any case. Our results indicate that TRPA1 has no major effect on mRNA transcription of these genes. Effects on translation, post-translational modification or degradation of target proteins that could explain the obtained results in 2D-GE may be present but have not been elucidated so far.

SM-affected proteins identified in our study are involved in several steps of gene transcription or mRNA translation. Caprin-1 is discussed to mediate the transport and translation of mRNAs of proteins involved in cell proliferation and migration in multiple cell types [45]. Striatin-4 binds calmodulin in a calcium-dependent manner and may function as scaffolding or signaling protein [46]. Nucleolin is a nucleolar phosphoprotein involved in fundamental aspects of transcription regulation, cell proliferation and growth [47]. It is thought to play a role in pre-rRNA transcription and ribosome assembly and in the process of transcriptional elongation [48]. *GPHN* and *FHL1* are proteins involved in organization of the cytoskeleton and protein-cytoskeleton interactions [49,50]. In addition, *FHL1* is involved in nuclear gene regulation processes [50]. *ELAVL1* is an RNA-binding protein that binds to the 3’-UTR region of mRNAs and increases their stability [51,52]. Only for *SFXN1*, a protein that might be involved in the transport of a component required for iron utilization into or out of the mitochondria [53,54], and *NOSIP*, a ubiquitin-protein ligase that negatively regulates nitric oxide production by inducing NOS1 and NOS3 translocation to actin cytoskeleton and inhibiting their enzymatic activity [55], a direct function in protein biosynthesis has not been described yet. Whether the identified proteins are indeed involved in the molecular toxicology of SM or related compounds has to be proven but is not part of this study.

Exposure of human keratinocytes with CEES (a monofunctional analog of SM) increased *HSPA6* levels [56]. In addition, CEES was identified as an activator of TRPA1 [4]. Results obtained in our study suggest a link between the expression of *HSPA6* and TRPA1 activation by alkylating compounds: SM increased *HSPA6* mRNA levels beginning 3 h after exposure, which was significantly prevented by AP18 pre-treatment (Figure 3A) and induced *HSPA6* protein formation after 24 h (Figure 1B). HEKwt cells did not respond to SM while human A549 lung epithelial cells, endogenously expressing TRPA1, revealed similar results with regard to *HSPA6* mRNA levels compared to HEKA1 cells (Figure 3B).

HSPs are molecular chaperones that regulate the folding, degradation and assembly of proteins [57]. After cellular stress such as intense heat, heavy metal exposure, UVB light, oxidative stress or inflammation, HSPs are up-regulated to protect cell proteins against aggregation [58,59]. *HSPA6* is especially responsible for the correct folding and activation of many proteins [60].

It is well known that SM exposure results in the formation of reactive oxygen species (ROS) or reactive nitrogen species in vitro [34,35]. ROS can induce protein damage, instability, aggregation and can even provoke cell death but have also been shown to induce HSPs [61]. Therefore, it is reasonable to assume that HSP induction may also be the consequence of SM-induced oxidative stress in our experiments.

It was previously postulated that elevation of [Ca^2+^]_i_ through TRP channel activation (in particular TRPA1 and TRPV1) activates the mitochondrial tricarboxylic acid cycle, which generates ATP as well as ROS [62,63]. In a study by Gould et al., CEES was proven to affect mitochondrial function in lung cells resulting in ROS formation [64]. Ray et al. demonstrated that SM induced an increase of [Ca^2+^]_i_ localized to mitochondria [65]. However, TRP channels were not considered as potential mediators of the observed phenomena.

Our work suggests a distinct role of TRPA1 beyond an immediate effect of SM on mitochondria. Inhibition of TRPA1 by AP18 is suggested to attenuate the SM-induced increase of [Ca^2+^]_i_ and thus, the generation of ROS and *HSPA6* subsequently. However, AP18 was insufficient to completely prevent *HSPA6* induction in our experiments indicating that TRPA1 is not exclusively responsible for *HSPA6* up-regulation. In addition to TRPA1-mediated ROS formation, SM-induced depletion of antioxidants, lipid oxidation or direct effects on mitochondria can cause severe oxidative stress which is independent of TRPA1 and may also trigger *HSPA6* expression [66,67,68].

**Figure 4:**
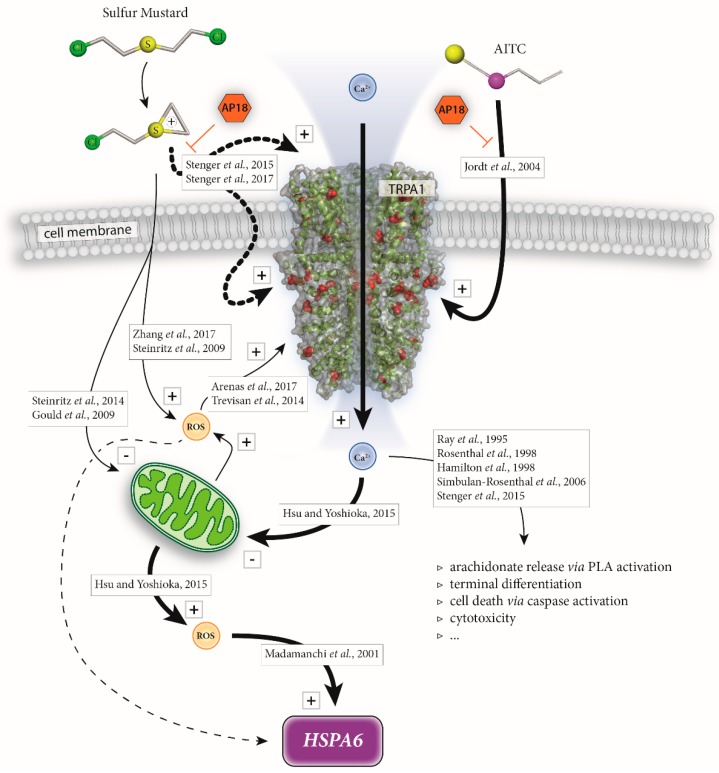
Schematic overview suggesting TRPA1-mediated induction of *HSPA6* after SM exposure. SM activates TRPA1 channels with a still unknown extra- or intracellular binding site (bold dotted lines). SM as well as AITC treatment increase intracellular Ca^2+^ ([Ca^2+^]_i_) levels. AP18 prevents both SM- and AITC-induced TRPA1 activation. [Ca^2+^]_i_ affects mitochondrial function thereby producing ROS which triggers *HSPA6* induction. SM may also cause ROS formation through disturbance of mitochondrial function or other yet not well-defined mechanisms thereby potentially contributing to *HSPA6* induction (dashed line) without involvement of TRPA1. Elevation of [Ca^2+^]_i_ also results in additional biological effects (open triangles). Text boxes list references describing the illustrated effects. Activation, increase or induction is marked with “+” while impairment of mitochondria is indicated with “−”.

Other biological effects of SM-induced increase of [Ca^2+^]_i_ such as phospholipase A activation and subsequent arachidonate release, terminal differentiation of human keratinocytes, induction of cell death through caspases, in addition to the above discussed Ca^2+^/ROS/*HSPA6* cascade, were described [4,65,69,70,71]. A synopsis depicting the interaction of cellular events after SM-exposure with focus on TRPA1 and *HSPA6* is given in Figure 4.

Our results suggest that SM causes an increase of [Ca^2+^]_i_ and subsequent induction of *HSPA6*, which is in part mediated by TRPA1 channels. Increase of ROS, presumably originating from mitochondrial stress, is a feasible cause for the observed *HSPA6* induction. An inhibition of TRPA1 in HEKA1 and A549 cells attenuated the SM-induced expression of *HSPA6* and thereby pointing to a distinct role of TRPA1 ion channels. Whether induction of *HSPA6* after SM exposure is a protective cellular defense mechanism as it may protect against stress-induced apoptosis [72] cannot be answered at this point and should be addressed in future research. Nevertheless, TRPA1 channels were proven to be part of the very complex molecular toxicology of SM and a step closer into the spotlight of SM research.

## Figures and Tables

**Table 1:** Protein assignment of differentially down-regulated protein spots. SM-induced and TRPA1-dependent down-regulated spots were analyzed by nanoHPLC-ESI MS/MS. Peptides were identified by NCBInr protein database search. Molecular weights (MW) were taken from UniProt database and theoretical pI values were calculated using isoelectric point calculators [26,27]. MS/MS scores represent sums over all MS/MS scores of every significant peptide.

	Gene Name	Protein Name	UniProt ID	MW[kDa]	pI[Calculated]	Number of Identified Peptides	MS/MS Score
Figure 1C	*CAPRIN1*	Caprin-1	Q14444	78.5	5.0	17	864
	*STRN4*	Striatin 4	Q9NRL3	81.3	5.1	13	787
	*NCL*	Nucleolin	P19338	76.6	4.5	10	443
	*GPHN*	Gephyrin	Q9NQX3	80.4	5.1	7	372
Figure 1D	*SFXN1*	Sideroflexin 1	Q9H9B4	35.9	9.4	10	689
	*FHL1*	Four and a half LIM domain protein 1	Q13642	38.0	9.2	9	592
	*NOSIP*	Nitric oxide synthase interacting protein	Q9Y314	33.7	9.1	11	554
	*ELAVL1*	ELAV like protein 1	Q15717	36.2	9.6	8	445

## Supplementary Materials

The following are available online at http://www.mdpi.com/2073-4409/7/9/126/s1, Figure S1. Representative 2D silver-stained gel electrophoresis of 600 μM SM-exposed HEKA1 cells. Figure S2. RT-qPCR measurements of relative HSPA6 mRNA levels in HEKA1 and HEKwt cells. Table S1. Fold change values of RT-qPCR.
