# Supplementary material for: Transient Receptor Potential Channel A1 (TRPA1) Regulates Sulfur Mustard-Induced Expression of Heat Shock 70 kDa Protein 6 (HSPA6) In Vitro
Source: Cells. 2018 Aug 31;7(9):126. doi: 10.3390/cells7090126 (PMC6162519; doi:10.3390/cells7090126)
Supplement: Supplementary file 1 [file cells-07-00126-s001.pdf]

Supplementary Material

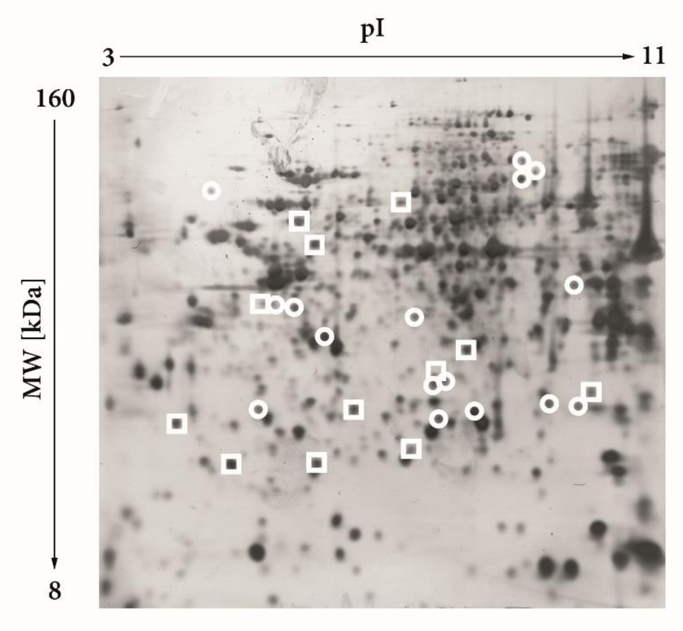

Figure S1. Representative 2D silver-stained gel electrophoresis of 600 μM SM-exposed HEKA1 cells. Isoelectric focusing of cell lysates was performed by 7 cm strips (pH 3–11, non-linear). Proteins were separated by 10% Bis-Tris gels. In total, 28 differential protein spots after SM exposure compared to controls were identified. White frames represent 12 up-regulated proteins while open white circles indicate 16 down-regulated protein spots. None of these proteins spots were affected by AP18 pre-incubation. Experiments were carried out with $n = 3$ per group. Molecular weight is indicated on the left and the pI value on top of the gels.

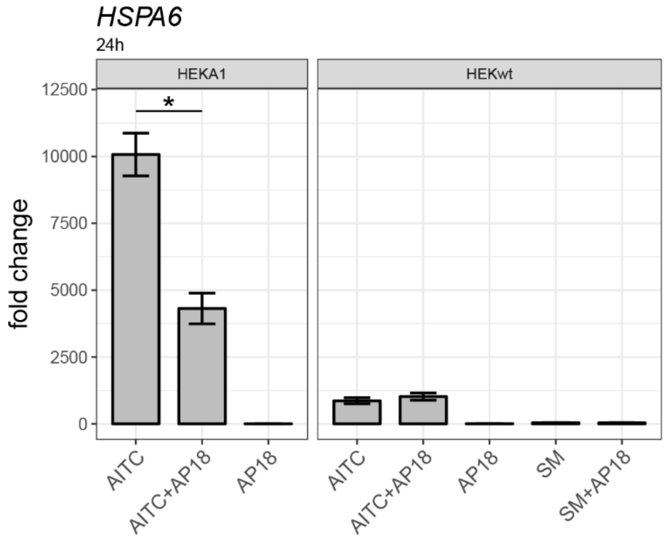

Figure S2. RT-qPCR measurements of relative *HSPA6* mRNA levels in HEKA1 and HEKwt cells. AITC treatment (25 μM) resulted in a significant change of *HSPA6* mRNA levels compared to unstimulated controls (normalized to 1.0, data not shown). Pre-incubation with AP18 (2 μM) attenuated the AITC effect significantly. AP18 alone had no effect. HEKwt cells responded with a small, rather unspecific increase of *HSPA6* gene expression that was not affected by AP18 pre-treatment. SM (600 μM) had no effect in HEKwt cells. Significant differences are marked by asterisk. Data are derived from 3 independent experiments ($n = 3$).

Table S1. Fold change values of RT-qPCR products in relation to controls (normalized to 1.0, data not included). Data are derived from 3 independent biological experiments (n = 3).

gene name	cell type	group	time [h]	fold change	lower 95 % CI	upper 95 % CI
CAPRIN1	HEKA1	SM	24	0,5	0,4	0,6
CAPRIN1	HEKA1	SM+AP18	24	0,5	0,4	0,5
ELAVL1	HEKA1	SM	24	0,5	0,4	0,6
ELAVL1	HEKA1	SM+AP18	24	0,4	0,3	0,4
FHL1	HEKA1	SM	24	0,8	0,6	1,0
FHL1	HEKA1	SM+AP18	24	0,7	0,6	0,8
GPHN	HEKA1	SM	24	0,5	0,4	0,5
GPHN	HEKA1	SM+AP18	24	0,4	0,4	0,4
HSPA6	A549	SM	24	6,8	6,5	7,2
HSPA6	A549	SM+AP18	24	5,0	4,7	5,3
HSPA6	HEKA1	AITC	24	10073,9	9273,6	10874,2
HSPA6	HEKA1	AITC+AP18	24	4314,6	3742,0	4887,1
HSPA6	HEKA1	AP18	1	2,2	1,9	2,4
HSPA6	HEKA1	AP18	3	2,2	1,9	2,4
HSPA6	HEKA1	AP18	5	2,2	1,9	2,4
HSPA6	HEKA1	AP18	24	2,2	1,9	2,4
HSPA6	HEKA1	SM	1	2,0	1,8	2,2
HSPA6	HEKA1	SM	3	9,5	8,2	10,8
HSPA6	HEKA1	SM	5	11,7	10,6	12,8
HSPA6	HEKA1	SM	24	16,0	14,4	17,6
HSPA6	HEKA1	SM+AP18	1	1,7	1,5	1,9
HSPA6	HEKA1	SM+AP18	3	6,7	6,1	7,4
HSPA6	HEKA1	SM+AP18	5	8,7	7,0	10,4
HSPA6	HEKA1	SM+AP18	24	9,8	8,9	10,7
HSPA6	HEKwt	AITC	24	863,1	748,1	978,0
HSPA6	HEKwt	AITC+AP18	24	1019,3	885,2	1153,9
HSPA6	HEKwt	AP18	24	5,2	4,5	5,9
HSPA6	HEKwt	SM	24	40,9	35,5	46,3
HSPA6	HEKwt	SM+AP18	24	39,5	31,9	47,1
NOSIP	HEKA1	SM	24	0,8	0,7	0,9
NOSIP	HEKA1	SM+AP18	24	0,5	0,4	0,6
NCL	HEKA1	SM	24	0,3	0,2	0,4
NCL	HEKA1	SM+AP18	24	0,2	0,2	0,3
SFXN1	HEKA1	SM	24	0,3	0,3	0,4
SFXN1	HEKA1	SM+AP18	24	0,3	0,2	0,3
STRN4	HEKA1	SM	24	1,4	1,1	1,7
STRN4	HEKA1	SM+AP18	24	1,2	1,0	1,4
